# ABCA4-associated disease in childhood and adolescence– a phenotype study

**DOI:** 10.1007/s00417-025-06884-9

**Published:** 2025-07-11

**Authors:** Jan-Philipp Bodenbender, Annekatrin Rickmann, Katarina Stingl, Susanne Kohl, Laura Kühlewein

**Affiliations:** 1 Centre for Ophthalmology, University Eye Hospital, University of Tübingen, Tübingen, Germany; 2 Knappschaftshospital Sulzbach, An der Klinik 10, 66280 Sulzbach, Saar Germany; 3 Molecular Genetics Laboratory, Institute for Ophthalmic Research, Centre for Ophthalmology, University of Tübingen, Tübingen, Germany

**Keywords:** Stargardt disease, ABCA4, Phenotype, Genotype, Optical coherence tomography, Fundus autofluorescence, Electroretinography, Childhood, Adolescence

## Abstract

*****What is known***:**

- So far, observational studies have primarily examined adults and have used these findings to define endpoints for interventional studies, which also serve as a reference for interventional studies in children
-  Most clinical trials currently use the measurement of definitely decreased autofluorescence

*****What is new***:**

- We have closely examined the variability of Stargardt disease in children
-  Our results suggest that the same inclusion criteria and endpoints used for adults should not be applied to children, e.g. definitely decreased autofluorescence is not suitable in pediatric cohorts because it has only a very low prevalence

**Supplementary Information:**

The online version contains supplementary material available at 10.1007/s00417-025-06884-9.

## Abstract (key message)

**Purpose:**

Aim of this study is to analyse the clinical picture of a pediatric *ABCA4*-related retinal disease cohort to evaluate possible inclusion criteria and outcome measures in children for future interventions.

**Methods:**

In this retrospective chart review study, cross-sectional data on the phenotype and genotype of patients under 18 years of age with *ABCA4*-associated retinal disease from the clinic for inherited retinal dystrophies at the University of Tuebingen were collected.

**Results:**

38 minor patients from 34 independent families with genetically confirmed *ABCA4*-associated disease were included in the study. Mean age was 10.3 years. 45 distinct genetic variants were observed. Thirteen patients had apparent homozygous *ABCA4* genotypes. The other carried two, three or four heterozygous *ABCA4* variants. More than half of the variants were missense variants (25/45, 55.6%). Mean best-corrected visual acuity was 0.80 logMAR. On optical coherence tomography, in 29 patients (78%) central photoreceptors were atrophic. Eight patients (22%) revealed a persistence of the central photoreceptor layers with visible external limiting membrane (ELM). In four of those, the ELM was markedly thickened. Only four patients revealed a definitely decreased autofluorescence in the macula.

**Conclusion:**

*ABCA4*-associated disease is already very heterogeneous in children, impeding the definition of reliable inclusion criteria for clinical trials. Although definitely decreased autofluorescence is a good endpoint in *ABCA4*-related disease trials, it is not suitable in pediatric cohorts due to its very low prevalence.

**Supplementary Information:**

The online version contains supplementary material available at 10.1007/s00417-025-06884-9.

## Introduction

Disease-causing variants in *ABCA4* are one of the most prevalent genetic causes of autosomal recessively inherited retinal dystrophies (IRD), and carrier frequency is at 1:20 [1]. The *ABCA4* gene encodes for the adenosine triphosphate-binding cassette transporter, alpha 4 subunit located in the rod and cone photoreceptor outer segments [1, 2]. When the ABCA4 transporter is dysfunctional, the membrane-trapped *all-trans*-retinal and N-retinylidene-phosphatidylethanolamine (NRPE) form components of lipofuscin (A2E and related bisretinoids) [3] with a toxic effect [4, 5]. The gene is located on chromosome 1p21 and comprises 128 kb of genomic sequence. There are more than 2,000 variants described in the literature (HGMD^®^professional database) [2], with missense variants being the most prevalent variant type [1]. Due to the high number of variants with variable pathogenic effect and the high number of possible allele combinations (i.e., genotypes), there is high variability in the phenotype and course of disease [1, 3, 6]. The most common clinical diagnosis associated with disease-causing variants in *ABCA4* is Stargardt disease (STGD1), a juvenile onset macular dystrophy and cone-rod dystrophy, but also cases with cone dystrophy, fundus flavimaculatus and retinitis pigmentosa have been reported [3].

Currently, there are no approved treatment options for STGD1 [2]. With 6.8 kb, the *ABCA4* coding sequence is too large to be carried by standard adeno-associated viruses [7]. Alternative vectors include dual AAV, lentiviral vectors and polymer-based nanoparticle delivery [8, 9]. Other approaches are the subretinal transplant of human embryonic stem cell derived retinal pigment epithelial (RPE) cells [10], or inhibition of retinol binding protein 4 (RBP4) or transthyretin (TTR) reducing the transport of vitamin A from the liver to the eye to decrease lipofuscin accumulation. Pharmaco-therapeutic experimental approaches include Tinlarebant (clinicaltrials.gov NCT05244304, NCT05266014) and STG-001 (clinicaltrials.gov NCT04489511) [11, 12]. Emixustat hydrochloride is a visual cycle modulator binding to RPE65 and preventing the production of 11-*cis*-retinal and A2E [12] (clinicaltrials.gov NCT03772665, NCT03033108). Avacincaptad pegol (Zimura) is an anti-C5 aptamer reducing complement system activity [12] (clinicaltrials.gov NCT03364153, NCT01367444). Another approach is the provision of deuterated vitamin A (ALK-001); here a deuterium atom impedes the dimerization of vitamin A and reduces production of A2E [12] (clinicaltrials.gov NCT02402660, NCT04239625, NCT02230228). Soraprazan is a reversible proton pump inhibitor that can break down accumulated toxic lipofuscin (EUDRA-CT: 2018-001496-20). None of these approaches have shown sufficient efficacy or were applied in clinical trial phases III for approval up to date.

There is probably only a small therapeutic window, as viable photoreceptors in the macula are only present in the early stage of disease [10]. Consequently, treatment in childhood would be desirable. Definition of readouts for therapies present a big challenge in interventional trials for *ABCA4*-related dystrophies. Firstly, there is large heterogeneity in the clinical presentation, which is especially problematic for systemic therapies needing a sham or placebo group in small cohorts of a rare disease. Secondly, objective readouts of progression are not well available. Electroretinographical findings can vary broadly among patients with STGD1 and have a relatively high intraindividual retest variability. Pupillometric readouts have a better sensitivity, but are not broadly available [13]. Morphological readouts which correlate with disease progression are not easily quantifiable. Quantitative autofluorescence, which has the potential to represent the increase in subretinal deposits, has turned to be very variable for a valid readout [14]. Additionally, the level of fundus autofluorescence does not behave linearly over the disease course; the normal fundus autofluorescence (FAF) is followed by pathologic hyperautofluroescence (e.g. flavimaculatus flecks) in the first stages of *ABCA4*-related disease and later with atrophy and thus local hypoautofluorescence. Therefore, most clinical trials currently use the measurement of definitely decreased autofluorescence (DDAF) in FAF as an inclusion criterion, since it is clearly definable and can only increase in the course of the disease. In this work, we have retrospectively analyzed the clinical picture of a pediatric *ABCA4*-cohort to evaluate the presence of such inclusion criteria and outcome measures.

## Methods

### Study design

The study was conducted in accordance with the Declaration of Helsinki, with approval from the ethics committee of the Medical Faculty of the University of Tübingen (project no. 138/2022BO2, 116/2015BO2, 349/2003V) and was registered in ClinicalTrials.gov under the study number NCT06377150. Data were evaluated retrospectively from patients who presented to the clinics for inherited retinal dystrophies at the Centre for Ophthalmology of the University of Tübingen, Germany, a tertiary referral center.

### Patients

Patients with proven or likely biallelic disease-causing variants in *ABCA4* who presented to our clinic between January 2014 and April 2021 and were younger than 18 years of age at the time of examination were included in the study. The data were collected during the patients’ initial presentation at the clinic.

### Ophthalmological examination

The patients’ ocular history was recorded. The ophthalmological examination included best-corrected visual acuity (BCVA) using a numeric eye chart measuring decimal visual acuity, which was transformed into the logarithm of the minimum angle of resolution (logMAR) for further analysis [15], slit lamp and dilated fundus examination, fundus photography (Topcon TRC-50IX, Topcon Corporation, Tokyo, Japan; Visucam 500, Carl Zeiss Meditec, Jena, Germany), and ultra-wide-field scanning ophthalmoscopy (Optos California Panoramic Ophthalmoscope P200DTx, Optos, Marlborough, MA, USA). Spectral domain optical coherence tomography (OCT) imaging was performed using Spectralis^®^ HRA + OCT (Heidelberg Engineering GmbH, Heidelberg, Germany). Fundus autofluorescence (FAF) imaging was performed using Spectralis^®^ HRA + OCT (Heidelberg Engineering GmbH, Heidelberg, Germany, *λ* = 488 nm, emission 500–700 nm, field of view 30° × 30° or 55° × 55°), or Optos California Panoramic Ophthalmoscope P200DTx (Optos, Marlborough, MA, USA). FAF findings were classified in three different patterns according to the literature [16, 17]: type 1 with a localized decreased FAF signal at the fovea and a homogeneous background, type 2 with a localized decreased FAF signal at the macula and a heterogeneous background and type 3 with multiple areas of decreased FAF signal at the posterior pole with a heterogeneous background [16]. In the case of centrally reduced autofluorescence, a distinction was made between definitely decreased autofluorescence (DDAF), where DAF was nearly 100% black, and questionably decreased autofluorescence (QDAF), where DAF was between 50 and 90% black. Borders of QDAF were assessed as well demarcated or poorly demarcated [18]. Full-field electroretinogram (ff-ERG) testing (Tabletop E3 console, Colordome, Espion software, Diagnosys, Lowell, MA, USA) was performed according to the International Society for Clinical Electrophysiology of Vision (ISCEV) standards [19]. ERG findings were classified as described previously [6, 20, 21]: patients in group 1 had normal photopic and scotopic responses, patients in group 2 had decreased photopic and normal scotopic responses, and patients in group 3 had decreased photopic and scotopic responses.

### Genetic testing and variant classification

All index patients received diagnostic genetic testing by IRD disease gene panel sequencing or *ABCA4* sequencing for known familial *ABCA4* variants in various diagnostic genetic laboratories and setups. One patient (ZD574) that was initially identified with a single heterozygous *ABCA4* nonsense variant in the diagnostic genetic setting was further analyzed by targeted *ABCA4* locus sequencing in a research setting [22].

Depending on the availability of DNA samples from additional family members, segregation analysis was performed either in a diagnostic genetic or research context by Sanger sequencing out of PCR amplified genomic DNA of the respective variant carrying exon(s) (Table 1).

**Table 1:** Age at first presentation, gender, clinical diagnosis, BCVA, ERG, and variants of all patients

Family Key	Age	Gender	Clinical diagnosis	BCVA [logMAR]	ERG - DA 0.01				ERG LA 3.0 flicker 31 Hz		Variants
					a - amplitude [µV]	a - implicit time [ms]	b - amplitude [µV]	b - implicit time [ms]	peak-to-trough - amplitude [µV]	peak-to-trough - implicit time [ms]	
RCD 702-2	5	f	maculopathy	0.00	7.27	42.00	282.40	99.00	63.84	6.00	c.214G > A;p.(Gly72Arg) c.214G > A;p.(Gly72Arg)
RCD 756	5	m	cone-dystrophy	1.30	−15.06	43.00	215.54	112.00	46.35	28.00	c.3701 C > T;p.(Pro1234Leu) c.3701 C > T;p.(Pro1234Leu)
MST 446*	6	f	cone-rod-dystrophy	0.60	−11.32	47.00	56.89	99.00	35.00	20.00	c.4849–2 A > G;p.(?) c.4849–2 A > G;p.(?)
MDS 329	6	m	cone-dystrophy	0.70	26.48	45.00	291.41	104.00	52.13	18.50	c.214G > A;p.(Gly72Arg) c.1622T > C;p.(Leu541Pro) c.3113 C > T;p.(Ala1038Val)
ZD 620*	7	f	cone-dystrophy	0.40	−78.00	33.00	268.68	69.00	37.40	16.00	c.1988G > A;p.(Trp663Ter) c.4918 C > T;p.(Arg1640Trp)
MST 229*	7	m	cone-rod-dystrophy	1.50							c.[5882G > A;5512 C > G]; p.[(Gly1961Glu); (His1838Asp)] c.[5882G > A;5512 C > G]; p.[(Gly1961Glu); (His1838Asp)]
MST 450-2	8	m	maculopathy	0.10							c.3364G > A;p.(Glu1122Lys) c.5714 + 5G > A;p.(?)
MST 329	8	m	maculopathy	0.60							c.634 C > T;p.(Arg212Cys) c.1622T > C;p.(Leu541Pro) c.3113 C > T < p.(Ala1038Val)
RCD 824*	8	f	cone-rod-dystrophy	0.90	−18.55	38.00	63.16	104.00	18.99	20.00	c.4254-1G > C;p.(?) c.6229 C > T;p.(Arg2077Trp)
RCD 790*	8	m	cone-dystrophy	1.00	−30.52	38.00	186.45	65.00	22.78	16.50	c.5917del; p.(Val1973Ter) c.5917del; p.(Val1973Ter)
MST 353-2*	9	m	maculopathy	0.80							c.571_580dup; p.(Gly194ValfsTer89) c.6077T > C;p.(Leu2026Pro)
RCD 704	9	f	maculopathy	0.90	−33.41	40.00	348.53	88.00	77.36	13.50	c.1807T > C;p.(Tyr603His) c.1807T > C;p.(Tyr603His)
ZD 574*	9	f	maculopathy	1.00	−0.77	48.00	171.18	88.00	65.38	14.00	c.5917delG; p.(Val1973Ter) c.5197-557G > T;p.(?)
RCD 697*	9	f	cone-dystrophy	1.00	−12.98	40.00	340.79	93.00	24.80	16.50	c.5018 + 2 T > C;p.(?) c.5018 + 2 T > C;p.(?)
RCD 702-1	9	m	cone-rod-dystrophy	1.20	−20.77	49.00	53.77	84.00	77.26	10.00	c.214G > A;p.(Gly72Arg) c.214G > A;p.(Gly72Arg)
RCD 674*	9	m	maculopathy	1.30							c.2933G > A;p.(Gly978Asp) c.4919G > A;p.(Arg1640Gln)
MST 450-1	10	F	maculopathy	0.60	−38.54	37.00	225.03	89.00	66.01	15.50	c.3364G > A;p.(Glu1122Lys) c.5714 + 5G > A;p.(?)
MST 435	10	F	maculopathy	0.70	−13.42	38.00	225.09	91.00	95.85	16.00	c.5318 C > T;p.(Ala1773Val) c.5318 C > T;p.(Ala1773Val)
MST 362*	10	F	maculopathy	0.80							c.768G > T;p.(Val256=) c.5461-10T > C;p.(?)
MST 440	10	f	cone-dystrophy	1.00	−13.55	40.00	252.42	91.00	37.17	21.00	c.2894 A > G;p.(Asn965Ser) c.2894 A > G;p.(Asn965Ser)
MST 422	10	m	cone-dystrophy	1.00	−11.29	40.00	328.08	95.00	46.65	14.50	c.1622T > C;p.(Leu541Pro) c.3113 C > T < p.(Ala1038Val) c.5461-10T > C;p.(?) c.5603 A > T;p.(Asn1868Ile)
MST 412	10	m	maculopathy	1.30	−31.48	35.00	325.42	80.00	79.61	15.50	c.4462T > C;p.(Cys1488Arg) c.4462T > C;p.(Cys1488Arg)
ZD 545^§^	10	f	cone-rod-dystrophy	1.30	−12.67	47.00	176.03	100.00	29.18	16.00	c.2588-7_2588-5delinsGG; p.(?) c.3259G > A;p.(Glu1087Lys)
MST 326*	11	f	maculopathy	0.40	−40.51	38.00	366.53	77.00	166.64	60.50	c.2041 C > T;p.(Arg681Ter) c.6079 C > T;p.(Leu2027Phe)
MST 414	11	f	cone-dystrophy	1.30	−32.91	35.00	271.06	84.00	70.82	8.00	c.2829del; p.(Pro944GlnfsTer6) c.2829del; p.(Pro944GlnfsTer6)
MST 426-2*	12	m	maculopathy	0.10	−38.65	40.00	179.93	74.00	49.60	8.00	c.3322 C > T;p.(Arg1108Cys) c.3329-1G > A;p.(?)
MST 411^§^	12	f	maculopathy	0.70	−59.05	43.00	439.91	85.00	112.94	15.00	c.1253T > C;p.(Phe418Ser) c.2626 C > T/p.(Gln876Ter)
MST 408*	12	m	maculopathy	0.70	−56.68	35.00	269.06	78.00	86.09	15.50	c.571-1G > T;p.(?) c.634 C > T;p.(Arg212Cys)
MST 353-1*	12	f	maculopathy	0.80	−4.91	37.00	443.64	81.00	60.41	13.50	c.571_580dup; p.(Gly194ValfsTer89) c.6077T > C;p.(Leu2026Pro)
MST 425	13	f	maculopathy	0.50	−20.12	35.00	325.63	86.00	84.00	20.50	c.3664_3669del; p.(Val1222_Glu1223del) c.5714 + 5G > A;p.(?)
RCD 718	13	m	cone-rod-dystrophy	1.30							c.3642_3644del; p.(His1215del) c.3642_3644del; p.(His1215del)
MST 407	13	f	maculopathy	1.30	−17.45	43.00	246.42	99.00	70.34	13.00	c.1903 C > T;p.(Gln635Ter) c.1411G > A;p.(Glu471Lys) c.5461-10T > C;p.(?) c.5603 A > T;p.(Asn1868Ile)
MST 356*	14	m	maculopathy	0.50	−46.95	39.00	279.76	82.00	98.51	14.50	c.5714 + 5G > A;p.(?) c.1957 C > T;p.(Arg653Cys)
MST 426-1*	14	m	maculopathy	0.70	−31.73	40.00	278.15	91.00	107.37	7.50	c.3322 C > T;p.(Arg1108Cys) c.3329-1G > A;p.(?)
RCD 827	14	f	cone-rod-dystrophy	1.30	2.50	41.00	169.01	65.00	37.37	13.50	c.1622T > C;p.(Leu541Pro) c.3113 C; p.(Ala1038Val) c.4234 C > T;p.(Gln1412Ter)
MST 428	15	F	maculopathy	0.10	−40.18	37.00	299.10	75.00	147.85	16.50	c.52 C > T;p.(Arg18Trp) c.5882G > A;p.(Gly1961Glu)
MST 441	16	f	maculopathy	0.20	−27.82	40.00	367.37	98.00	111.57	14.50	c.1622T > C;p.(Leu541Pro) c.3113 C > T;p.(Ala1038Val) c.5882G > A;p.(Gly1961Glu)
MST 415*	16	m	cone-rod-dystrophy	0.40	−30.84	36.00	189.57	76.00	49.04	13.00	c.4234 C > T;p.(Gln1412Ter) c.5714 + 5G > A;p.(?)

*: segregation analysis in both parents confirmed biallelic inheritance; §: one variant was displayed heterozygously in one parent, while the other variant was excluded; BCVA = best-corrected visual acuity, ERG =, DA = dark-adapted, LA = light-adapted, f = female, m = male; normal values (median [min, max]): DA 0.01: a-wave: amplitude: −32.12 [−43, −21], implicit time: 34.00 [33, 37 ]; b-wave: amplitude: 382.88 [285, 554], implicit time: 81.50 [70, 92]; LA 3.0 flicker 31 Hz: peak-to-trough: amplitude: 144.20 [95, 204], implicit time: 26.50 [22, 28 ]

Variant nomenclature of the *ABCA4* variants adheres to HGVS variant description recommendations (http://varnomen.hgvs.org/) and was based on NCBI reference sequence (NM_000350.3, GRCh38,hg19 NC_000019.9 NG_009759.1 genome assembly) and Ensembl gene and transcript reference sequence ENST00000370225.4 comprising 50 coding exons.

Variant classification within this study was taken from the diagnostic genetic test reports and was reevaluated according to Cornelis et al. [26] and the ClinGen recommendations. Minor allele frequencies were retrieved from the Genome Aggregation Database (gnomAD) (v2.1.1) (https://gnomad.broadinstitute.org/).

### Data analysis

Data analysis was performed with SPSS (IBM SPSS Statistics for Windows, Version 28.0., released 2021, IBM Corp, Armonk, New York, USA). A p-value of less than 0.05 was considered statistically significant.

## Results

### Clinical findings

38 patients with genetically confirmed *ABCA4*-associated disease (i.e., macular, cone-, or cone-rod dystrophy) were included in the study. 55% (21/38) were female. The mean age of the patients at examination was 10.3 years (median 10.0, range 5–16 years). Ophthalmological findings were highly symmetrical in both eyes. Findings of the right eye are reported in the following. Mean BCVA was 0.80 logMAR (median 0.80 logMAR, range 0–1.50 logMAR, Table 1).

**Fig. 1:**
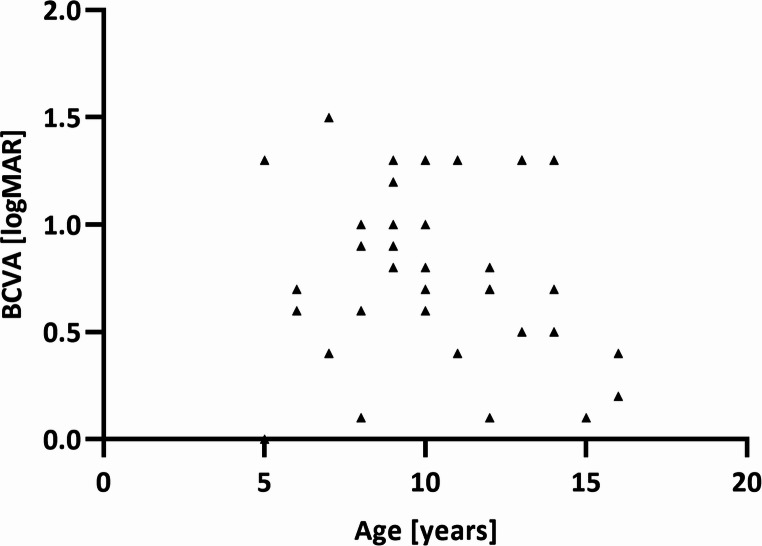
Age and BCVA (right eye) of 38 patients with genetically confirmed *ABCA4*-associated disease. Note that there was no statistically significant correlation between age and BCVA

In our cohort, there was no statistically significant correlation between age and BCVA (non-parametric Spearman correlation, *r* = −0.1361, *p* = 0.4153, *n* = 38, Fig. 1).

OCT imaging was performed in 37 patients (Supplementary Figure S1). In 29 patients (78%), central photoreceptors were atrophic. Eight patients (22%) revealed a persistence of the central photoreceptor layers with visible external limiting membrane (ELM). In four of those, the ELM was markedly thickened (e.g. MST450-2). In one of those, the central ellipsoid zone (EZ) line was not visible (MST415). Mean BCVA of patients without visible ELM was 0.92 logMAR (median 0.90 logMAR, range, 0.40–1.50 logMAR), whereas mean BCVA of patients with visible ELM was 0.35 logMAR (median 0.15 logMAR, range, 0.00–1.30 logMAR). This difference was statistically significant (*p* = 0.0007, Mann-Whitney U = 31.50).

**Fig. 2:**
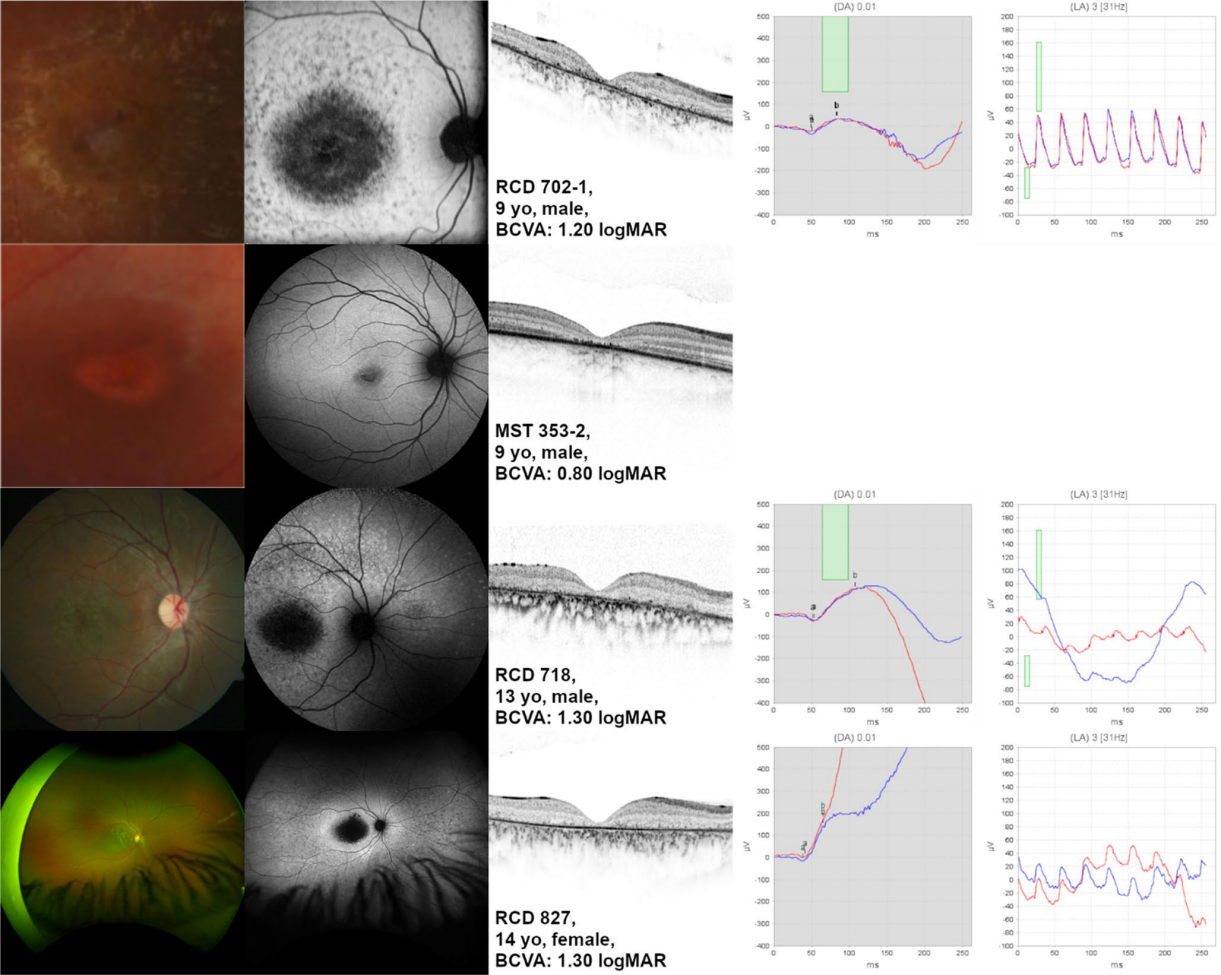
Definitely decreased fundus autofluoresence (DDAF) in childhood and adolescent ABCA4-related retinal dystrophy. Note that only four of the patients in our cohort revealed a DDAF-lesion

FAF imaging was performed in 33 patients (Supplementary Figure S1). Thirteen patients had a type 1 pattern, and 18 patients a had type 2 pattern. No patient had a type 3 pattern. One patient (MST 415) revealed no abnormalities at the posterior pole except for flecks nasal to the optic disc. Another patient (MST 450-2) showed increased autofluorescence at the posterior pole. Of the 31 patients with a localized decreased FAF signal at the macula, FAF was rated as QDAF in 27 patients, of which the borders were assessed as well demarcated in 13 and poorly demarcated in 14. In four patients only, FAF was assessed as DDAF. Three of these patients revealed multiple central DDAF lesions with a total size of 5.80 mm² (RCD 827), 7.68 mm² (RCD 702-1) and 9.69 mm² (RCD 718), respectively (Fig. 2). Each of these patients had a BCVA of 1.20 logMAR or lower. Another patient (MST 353-2) revealed a central single DDAF lesion of 0.22 mm² and a BCVA of 0.80 logMAR.

Full-field-ERG was performed in 31 patients (Supplementary Figure S1). Seventeen patients (55%) had both normal scotopic and photopic responses consistent with a macular dystrophy (group 1), eight patients (26%) had normal scotopic but decreased photopic responses consistent with a cone dystrophy (group 2), and six patients (19%) had decreased scotopic and photopic responses consistent with a cone-rod-dystrophy (group 3). In these six patients the b-wave was more drastically affected compared to the a-wave.

Patients with macular dystrophy demonstrated a mean visual acuity of 0.62 logMAR. A continuous external limiting membrane (ELM) was observed in four of 17 patients (24%). Of 15 patients with FAF imaging, eleven showed a well demarcated questionably decreased autofluorescence (WDQAF) pattern (73%), four a poorly demarcated questionably decreased autofluorescence (PDQAF) pattern (27%), and none a DDAF pattern.

Patients with a cone dystrophy had a mean visual acuity of 0.96 logMAR. A continuous ELM was present in one of eight cases (13%). Of seven patients with FAF imaging, two showed a WDQAF pattern (29%), five a PDQAF pattern (71%), and none a DDAF pattern.

Patients with cone rod dystrophy had a mean visual acuity of 0.95 logMAR. A continuous ELM was present in one of six cases (17%). Of six patients with FAF imaging, one showed a WDQAF pattern (17%), two a PDQAF pattern (33%), and two a DDAF pattern (33%).

Patients with cone dystrophy or cone-rod dystrophy exhibited poorer visual acuity compared to those with macular dystrophy. Similarly, a higher number of patients in this group showed a disrupted ELM and poorly defined questionably decreased or even a definitely decreased autofluorescence.

No association between the phenotype and different variants were observed.

### Siblings

**Fig. 3:**
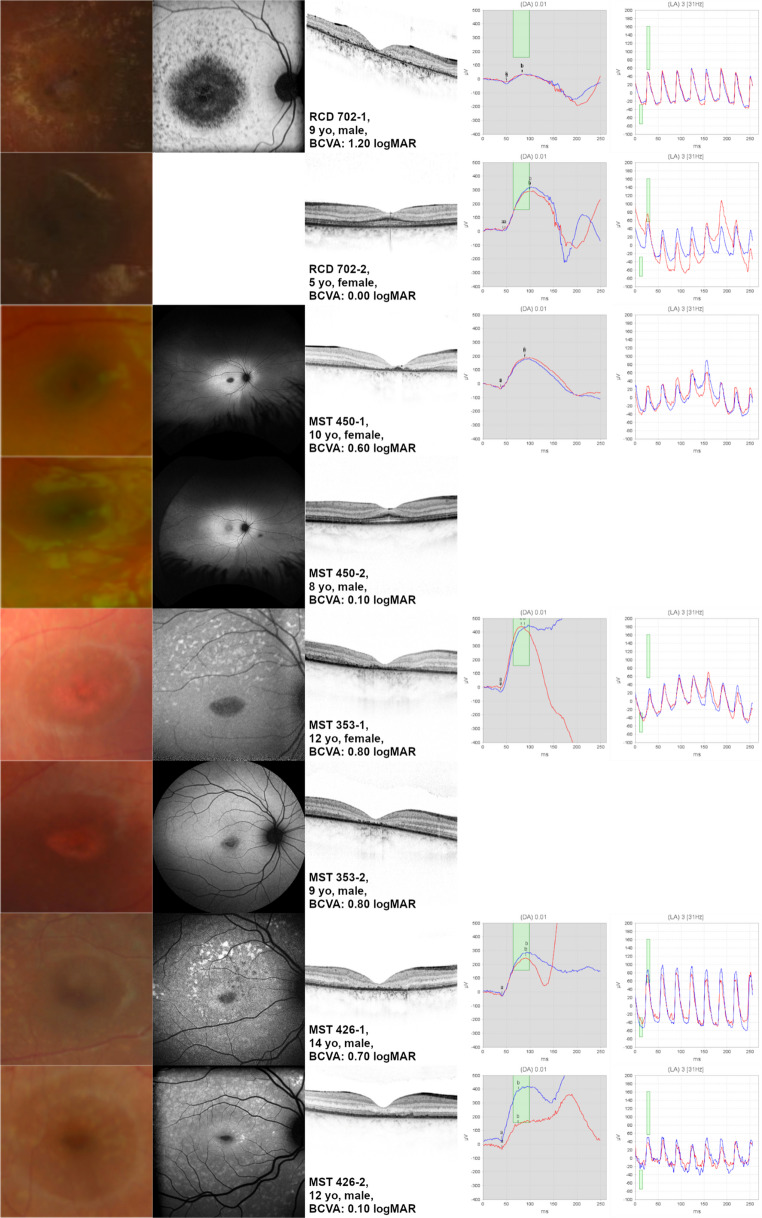
Findings of eight siblings included in the study

Eight siblings from four families were included in the study. Except for one sibling pair (MST 353), the findings were worse in the older sibling compared to the younger one (Table 2; Fig. 3).

**Table 2:** Findings of eight siblings included in the study

ID	Age [years]	Gender	Clinical diagnosis	BCVA [logMAR]	OCT centrally	FAF
RCD 702-1	9	m	cone-rod-dystrophy	1.20	atrophy of the outer retinal layers	DDAF (type 2)
RCD 702-2	5	f	maculopathy	0.00	reduced ONL thickness paracentrally, thickened ELM, continuous EZ line	not available
MST 450-1	10	f	maculopathy	0.60	atrophy of the outer retinal layers	WDQDAF (type 2)
MST 450-2	8	m	maculopathy	0.10	reduced ONL thickness paracentrally, thickened ELM, continuous EZ line	increased AF centrally
MST 353-1	12	f	maculopathy	0.80	atrophy of the outer retinal layers	WDQDAF (type 2)
MST 353-2	9	m	maculopathy	0.80	atrophy of the outer retinal layers	DDAF (type 1)
MST 426-1	14	m	maculopathy	0.70	atrophy of the outer retinal layers	PDQDAF (type 2)
MST 426-2	12	m	maculopathy	0.10	reduced ONL thickness, persistent ELM, absent EZ line	WDQDAF (type 2)

BCVA = Best-corrected visual acuity, OCT = optical coherence tomography, FAF = fundus autofluorescence, ONL = outer nuclear layer, ELM = external limiting membrane, EZ = ellipsoid zone, DDAF = definitely decreased autofluorescence, WDQDAF = well demarcated questionably decreased autofluorescence, PDQDAF = poorly demarcated questionably decreased autofluorescence, f = female, m = male

### Genetic findings

Forty-five (45) distinct variants were observed in this cohort of 38 juvenile *ABCA4*-IRD patients from 34 independent families including four pairs of siblings (Table 1). Age at genetic testing and confirmation of molecular diagnosis ranged from 3 to 17 years (mean and median 11 years of age). Thirteen patients were (apparent) homozygous. The other carried two, three or four heterozygous *ABCA4* variants, some expected to occur as commonly known complex alleles (e.g., c.[1622T > C;3113 C > T]). The most commonly observed variants were c.634 C > T;p.(Arg212Cys), c.1622T > C;p.(Leu541Pro), c.3113 C > T;p.(Ala1038Val), and c.5714 + 5G > A;p.(?) with five patients carrying these each (Supplementary Table S1). More than half of the variants were missense variants (25/45, 55.6%) which is consistent with the expected mutation spectrum. Additionally, five nonsense variants, 10 variants known or predicted to cause missplicing, one small duplication and four small deletions were identified. Of the latter, one results in frame-shift and premature termination codon, one in a direct premature termination codon and two are in-frame deletions of one and two encoded amino acids, respectively.

Segregation analysis was possible in both parents in 15 cases (Table 1), in six of which also additional affected siblings were tested positive for the disease-associated genotype, confirming biallelic zygosity of the respective *ABCA4* variants. In two cases, single parents were tested, and in three cases, segregation analysis was possible in additional affected siblings but not the parents.

## Discussion

In this retrospective study, we analyzed the phenotype and genotype of 38 patients with *ABCA4*-associated disease that were younger than 18 years old at the time of examination. To our knowledge, this is one of the largest cohorts of young and adolescent patients with *ABCA4*-associated disease published to date. Comparable studies evaluating the natural history of *ABCA4*-accociated disease are the ProgStar study with a retrospective cohort, including 251 patients with disease-causing variants in *ABCA4* and a typical STGD1 disease phenotype of which 60 were younger than 18 years old at the time of examination [27], and a prospective cohort, including 259 patients (with 45 patients younger than 18 years) [27], and a study by Khan et al., including eight children with biallelic variants in *ABCA4* without macular atrophy [10].

### Clinical findings

#### BCVA

In the retrospective part of the ProgStar study [28], mean BCVA was 0.8 logMAR just like in our study. However, only 24% (60/251) of the patients that were included in the retrospective part of the ProgStar study were younger than 18 years of age. One might argue that this similarity to our study can be explained by the plateau which STGD1 patients can reach without further deterioration of BCVA later in life. However, 27% of the eyes in our study had severe visual impairment to blindness, compared to only 13% in the retrospective part of the ProgStar study. It may be that there was a bias towards including milder cases in the ProgStar studies, of which the inclusion criteria were different from our study. Thus, the presence of DDAF (which was a major inclusion criterion for both the retro- and the prospective parts of the ProgStar studies) does not always reflect more advanced disease. We saw no statistically significant correlation between age and BCVA. This is in accordance with the findings of other studies describing the loss of visual acuity in *ABCA4*-associated disease as highly variable [29]. However, most of the patients in our cohort revealed a BCVA of < 0.5 logMAR, consistent with a rapid decline in BCVA in patients with an early onset under 15 years of age [28].

### FAF

In our cohort, 18 patients (47%) revealed widespread hyperautofluorescent flecks (type 2). In comparable studies with patients 6 years and older, the proportion of patients with type 2 was higher (60%, 41 of 68 patients) [17].

In only four of our patients, the central decreased FAF was assessed as DDAF, the youngest patient being 9 years of age. ProgStar study patients had DDAF at the initial visit in 58% (224 of 458) [30] and in 74% (184 of 390) of enrolled eyes [25]. If only patients under the age of 18 were considered, DDAF was seen in 20% (19 of 94) [25]. In a cohort of young patients, it is important to differentiate decreased autofluorescence from physiological macular hypoautofluorescence, which is caused by luteal pigments [2, 31]. In Khan’s cohort, all patients revealed this central physiologic hypoautofluorescence, reduced in size [10], which makes the decrease of the physiologic hypoautofluorescence a potential parameter in early stages of disease. This was not seen in our cohort. Of note, Khan described numerous tiny hyperautofluorescent dots at the fovea in early disease in three patients [10]. In our cohort, only one patient with a very good BCVA revealed hyperautofluorescence in the foveal area, however more diffuse and not dot-like.

#### OCT

The first OCT sign in *ABCA4*-associated disease is a parafoveal reduction of the outer nuclear layer (ONL), which spares the foveola and results in a focal collapse of the inner retinal layers [10]. However, this is difficult to note and is only seen in very early stages of the disease. In our pediatric cohort, it was seen in two patients (RCD 702-2, MST 450-2) who were both younger siblings of diagnosed older siblings and both were asymptomatic.

In contrast, the thickening of the ELM in early stages of disease is more striking. It is supposed to be caused by atrophy of cones and a subsequent hypertrophy and migration of Müller cells [10, 32]. With disease progression, the ELM decreases until the ELM disappears completely [10]. As can be seen in our study, as well, the state of the ELM correlates well with functionality.

Reduced intensity of the ellipsoid zone (EZ) is supposed to be caused by disturbed photoreceptors, making it a good parameter in early stages of the disease [10, 32]. EZ loss is described as 1.6-fold greater than RPE atrophy [16]. Explanatory approaches either assume that photoreceptor degeneration precedes RPE loss in *ABCA4*-associated disease, or that functionally disturbed, but structurally intact RPE cells cause a functional defect of the photoreceptors and finally their demise [16, 20, 29]. Either way, loss of EZ intensity indicates retinal damage earlier than structural changes in the RPE. In our cohort, the EZ line was only visible in three patients, suggesting that EZ also disappears earlier than ELM, which does not represent neurological tissue [33]. As mean BCVA of all patients with a persistent EZ was higher than that of patients with a centrally atrophic EZ, it is also likely a good surrogate parameter for functionality.

Total ONL atrophy occurs later during the course of the disease, starting perifoveally [10], and it seems to be the most important structural parameter for visual acuity. In our study, we saw a significantly higher BCVA in patients without ONL atrophy compared to those with ONL atrophy. This is in line with Khan’s findings who rate ONL thickness as the current biomarker of choice [10] and it is also in line with the findings of the OCT part of the ProgStar study by Strauss et al., who found that the total macular volume in patients with STDG1-disease decreases continuously during the disease process [34].

#### ERG

Ff-ERG is an essential tool for patients with *ABCA4*-associated disease as it allows to distinguish the patient’s phenotype between *ABCA4-*associated macular dystrophy, cone dystrophy, and cone-rod dystrophy. Main findings in our cohort comprised that photopic ERG responses were more severely affected, scotopic ERG responses varied and the b-wave was more drastically affected compared to the a-wave. Khan also reported a low b-to-a-ratio or even electro-negative waveforms locating retinal dysfunction to a reduced activation of bipolar cells [10].

### Genetic findings

Early onset and/or severe *ABCA4*-related IRD are expected to result from the presence of two non-functional alleles [23, 35]. It is difficult to predict the functional consequence of missense variants, but nonsense and frame-shifting variants likely act as non-functional alleles [23, 35, 36]. Yet only one of our patients (RCD790) was homozygous for a nonsense variant, while all other patients carried at least one missense variant. Cornelis and coworkers proposed a severity catalog for *ABCA4* variants according to the allele frequency of these variants in cases and controls [26], and recently, Fenner and coworkers correlated age of onset versus genotype to assess severity of several common variants [37]. In our cohort of 38 juvenile and adolescent *ABCA4*-related IRD patients, 62% (28/45) variants were categorized as severe according to Cornelis and coworkers, one as moderate to severe and nine as moderate, further supporting the pathogenic effect of the variants identified in the patients of our study (Supplementary Table S1). If we compare the individual genotypes, 12 of the patients had an severe/severe, 14 an moderate/severe and only 9 an moderate/moderate genotype. The relatively high presence of rather severe variants is in concordance with early clinical symptomatic, leading to a manifest pediatric *ABCA4*-associated disease.

When patients are categorized according to the severity groups for *ABCA4* variants proposed by Cornelis et al. [26], the following clinical findings were observed. One patient with a moderate/mild genotype showed a visual acuity of 0.10 logMAR; the ELM was continuous on OCT, and ERG revealed normal scotopic and photopic responses. Five patients with a moderate/moderate genotype revealed a visual acuity of 0.88 logMAR; only one patient showed a continuous ELM on OCT, two patients revealed a maculopathy, one patient a cone dystrophy, and one patient a cone rod dystrophy. Nine patients with moderate/severe genotype had a visual acuity of 0.68 logMAR, three patients showed a continuous ELM on OCT, six patients revealed a maculopathy, one a cone rod dystrophy. Twelve patients with severe/severe genotype had a visual acuity of 0.93 logMAR, all patients revealed a central atrophy on OCT, five patients revealed a maculopathy, four patients a cone dystrophy, one patient a cone rod dystrophy.

A more severe disease manifestation might be expected in patients carrying variants classified as severe; however, this was not reflected in visual acuity. Nevertheless, none of the patients with variants classified as severe/severe exhibited a preserved ELM, and cone dystrophies were more frequently observed in this group.

Five patients harbored the complex allele c.[1622T > C;3113 C > T], in which c.1622T > C;p.(Leu541Pro) is classified as severe and c.3113 C > T;p.(Ala1038Val) as mild, in combination with various counter-alleles. Notably, the patient (MST441) carrying a mild counter-allele demonstrated markedly better visual acuity (0.20 logMAR) compared to the other patients, despite being of older age. This individual also exhibited a continuous ELM and normal photopic and scotopic responses on ERG. These findings suggest that, in patients with the complex allele, the phenotypic expression is primarily influenced by the severity of the counter-allele.

Two patients carried the complex allele c.[5461-10T > C;5603 A > T], in which c.5461-10T > C;p.(?) is classified as severe and c.5603 A > T;p.(Asn1868Ile) as mild, one in combination with an allele classified as severe, and the other with the complex allele c.[1622T > C;3113 C > T]. Both patients exhibited markedly reduced visual acuity and central atrophy on OCT.

If we maintain our assumption that the phenotype in a genotype involving the complex allele c.[1622T > C;3113 C > T] is primarily determined by the counter-allele, the pronounced disease manifestation in the patient with the complex counter-allele c.[5461-10T > C;5603 A > T] supports classifying the complex allele as severe.

Five patients could not be clearly assigned according to the categorization by Cornelis et al. [26].

As modifying factors are supposed to have an equal impact on the phenotype as the *ABCA4* variants themselves and most patients with *ABCA4*-associated retinal disease have a unique genotype, the clinical findings are considered to be more predictive for the course of the disease than the genotype [37]. This is reflected in the inclusion criteria of most clinical studies which require variants in the *ABCA4* gene, but without specific prerequisites in terms of severity.

### Clinical trials

Twenty-five interventional studies are currently (February 2024) listed on clinicaltrials.gov for STGD1, and one further study is listed in the EU Clinical Trials Register. Of these, two are phase 3 studies. The active substances are administered intravitreally, subretinally, parabulbarly, or orally.

#### Inclusion criteria for clinical trials

Inclusion criteria of listed clinical trials with intervention comprise a typical STGD1 appearance of the retina showing bilateral central maculopathy, fundus flecks or macular atrophy secondary to STGD1. Most trials request variants in the *ABCA4* gene, eight trials request two variants, six trials at least one variant, and one further study requests a “known genotype or genotype under study”. All other trials only request a clinical diagnosis. Besides, the inclusion criteria are primarily functionally oriented requiring a minimal BCVA mostly of 20/200, sometimes also explicitly an even worse visual acuity. Morphologically, the focus is primarily on fundus autofluorescence demanding an area of significantly reduced autofluorescence. Eight of the listed studies include children, with no difference in inclusion criteria between studies with children and adults. Atrophy is usually required as an inclusion criterion, since it is a suitable outcome measure for clinical trials as corresponding areas of decreased autofluorescence enable a highly reliable qualitative and quantitative grading [18, 25]. However, this renders the determination of inclusion criteria for exclusive pediatric cohorts a difficult task.

#### Outcome measures

Primary outcome measures of listed clinical trials are a change in BCVA and macular atrophy measured by change in decreased autofluorescence (DAF). A different measurement of change in autofluorescence was used in the Soraprazan study with change in mean quantitative autofluorescence value of an 8-segment ring centered on the fovea (qAF8), however, there was a high variability of this measure [14]. Two studies focus on the change of the area of EZ defect on OCT. The change in ERG is measured in three studies.

A potential outcome measure might be the volume of the entire retina in the central ring of the EDTRS grid. A clear correlation between the atrophy of the central photoreceptors and BCVA has been shown. As the inner retinal layers collapse over atrophic photoreceptors [10], the volume of the total retina correlates with the ONL volume. Measuring only the ONL or substructures like the EZ line and ELM, on the other hand, is more difficult and offers a reduced comparability as these are relatively heterogeneous.

### Strengths and limitations of the study

Strengths of the study include the large sample size for a pediatric *ABCA4*-associated disease cohort, the availability of various examination modalities for each patient, despite the fact that these were not acquired in a prospective setting, and the availability of confirmatory genetic results for all patients.

Limitations include the retrospective design of the study, causing heterogeneity of the patients’ data. The patients were not examined under trial conditions, e.g. a numeric eye chart was used for BCVA assessment. For fundus autofluorescence, different devices were used, so that measures of DDAF may vary. Our study is based on cross-sectional data lacking longitudinal data, which could be analyzed in a further study to draw conclusions about the clinical progress in a pediatric *ABCA4*-associated disease cohort.

## Conclusion

We have analyzed a large pediatric *ABCA4*-associated disease cohort and shown that the disease is already very heterogeneous in children. This makes it difficult to specify reliable inclusion criteria for studies that enable meaningful recruitment. Although DDAF is a good endpoint in *ABCA4*-related disease trials, it is not suitable in pediatric cohorts because it has only a very low prevalence, in our cohort only in four patients. Therefore, further studies are needed to shed more light on inclusion criteria in pediatric cohorts, whose treatment is likely to be the most promising due to not yet existing atrophy in the early stage of disease.

## Electronic supplementary material

Below is the link to the electronic supplementary material.

Supplementary Material 1

## Data Availability

The data presented in this study is contained within the article and supplementary material.

## Declarations

### References ABCA4 variants and classification table (Supplementary table S1)

[20, 22–24, 26, 36, 38–62].
