# Supplementary material for: ABCA4-associated disease in childhood and adolescence– a phenotype study
Source: Graefes Arch Clin Exp Ophthalmol. 2025 Jul 11;263(10):2747–61. doi: 10.1007/s00417-025-06884-9 (PMC12583354; doi:10.1007/s00417-025-06884-9)
Supplement: Supplementary file 1 — Supplementary Material 1 [file 417_2025_6884_MOESM1_ESM.docx]

**Supplement**

**Supplementary Figure S1: Clinical findings of the patients in this study (right eyes)**. Fundus imaging, Fundus autofluorescence, Optical coherence tomography, and ERG responses are shown. As for the ERG, the dark-adapted 0.01 single flash–response is shown to display rod responses, and the 31Hz flicker-response is shown to display cone responses.


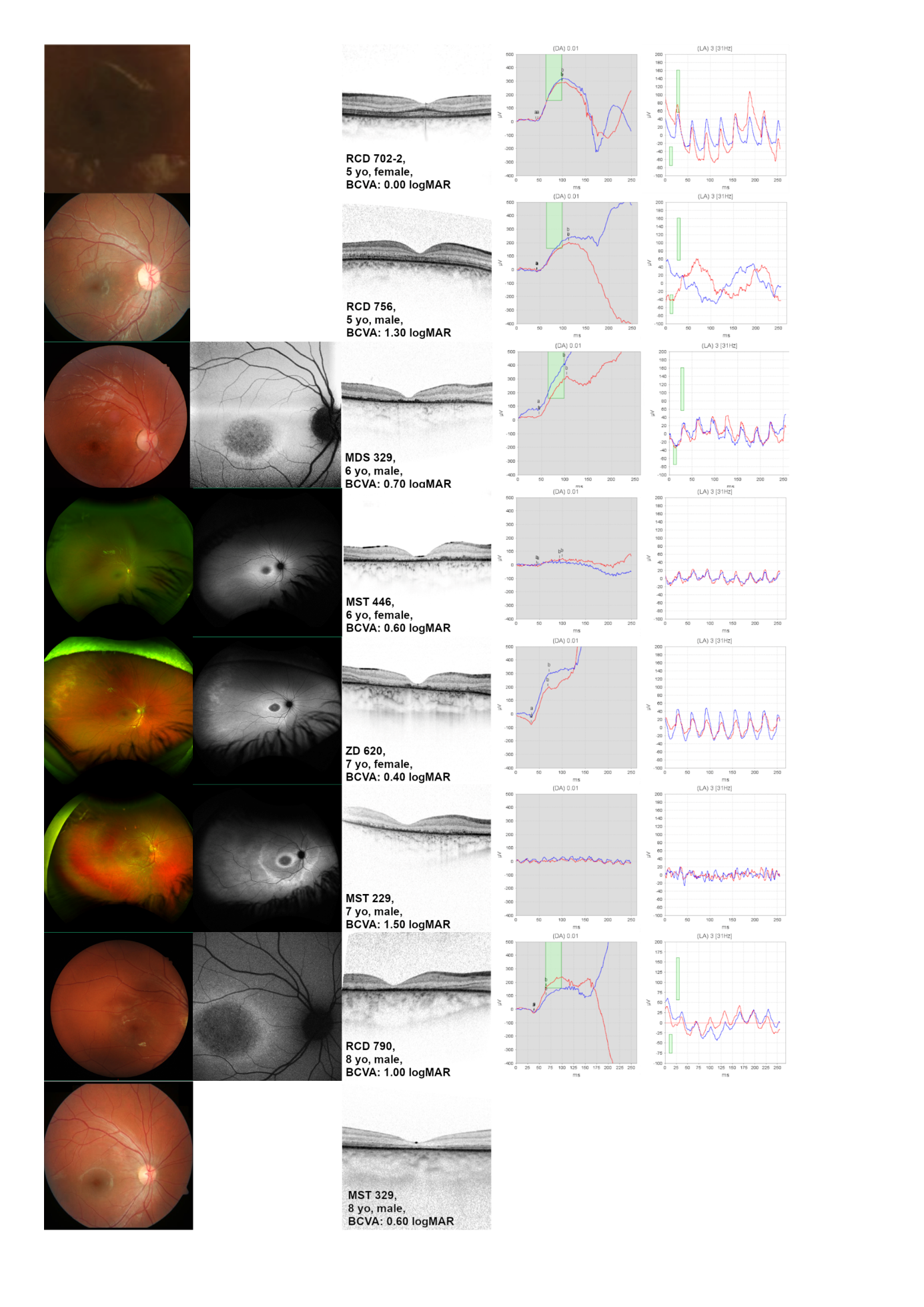


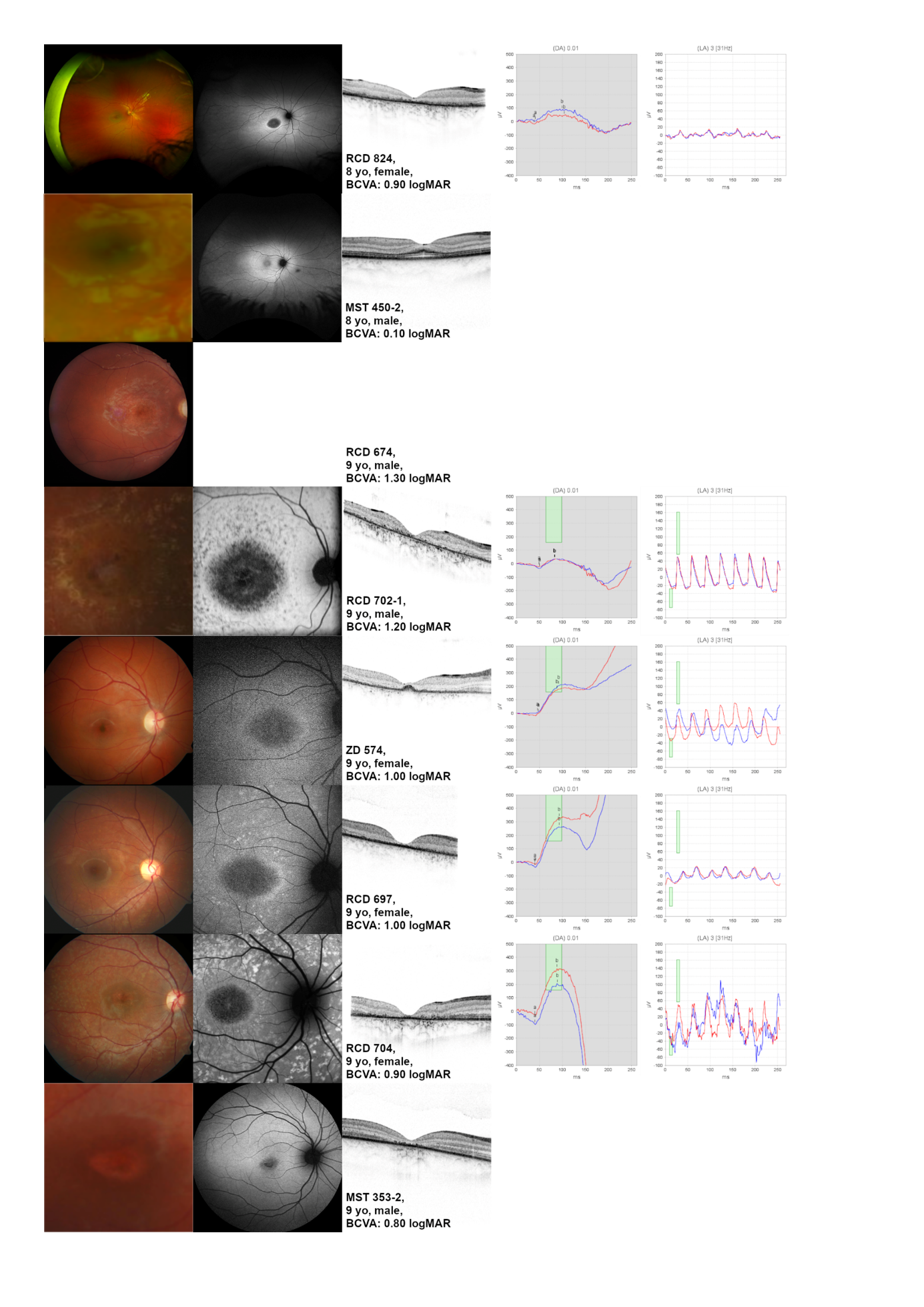


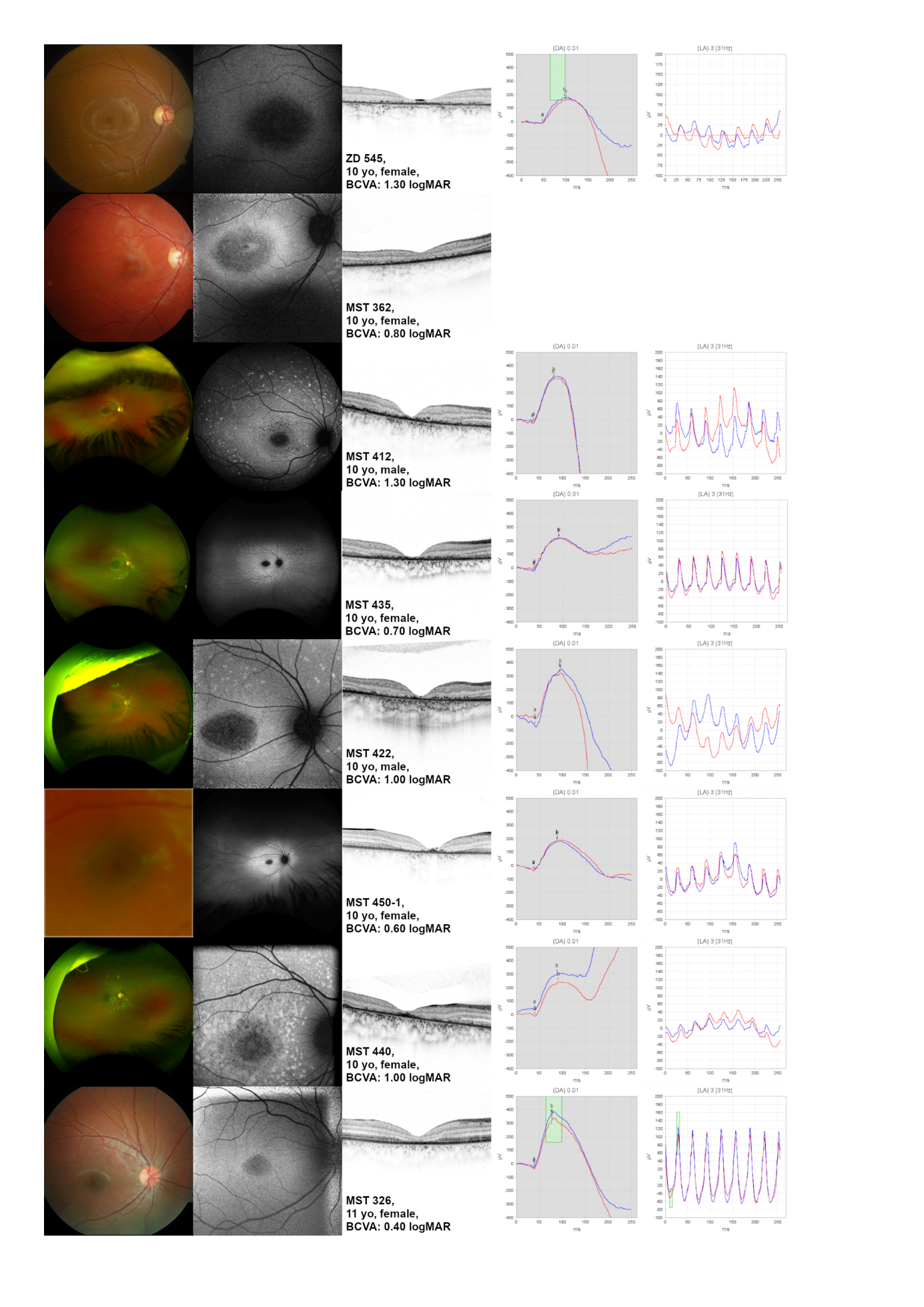


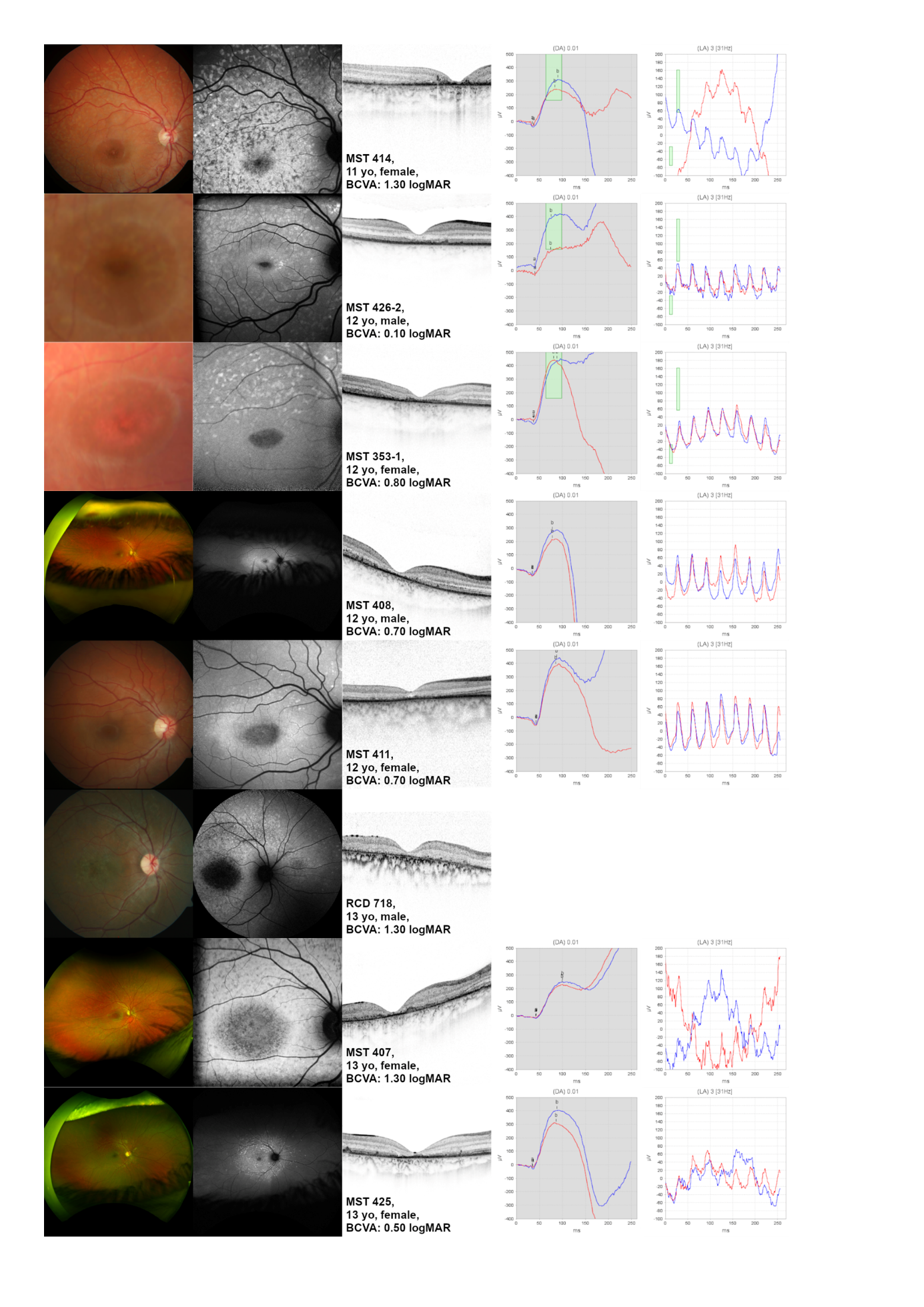


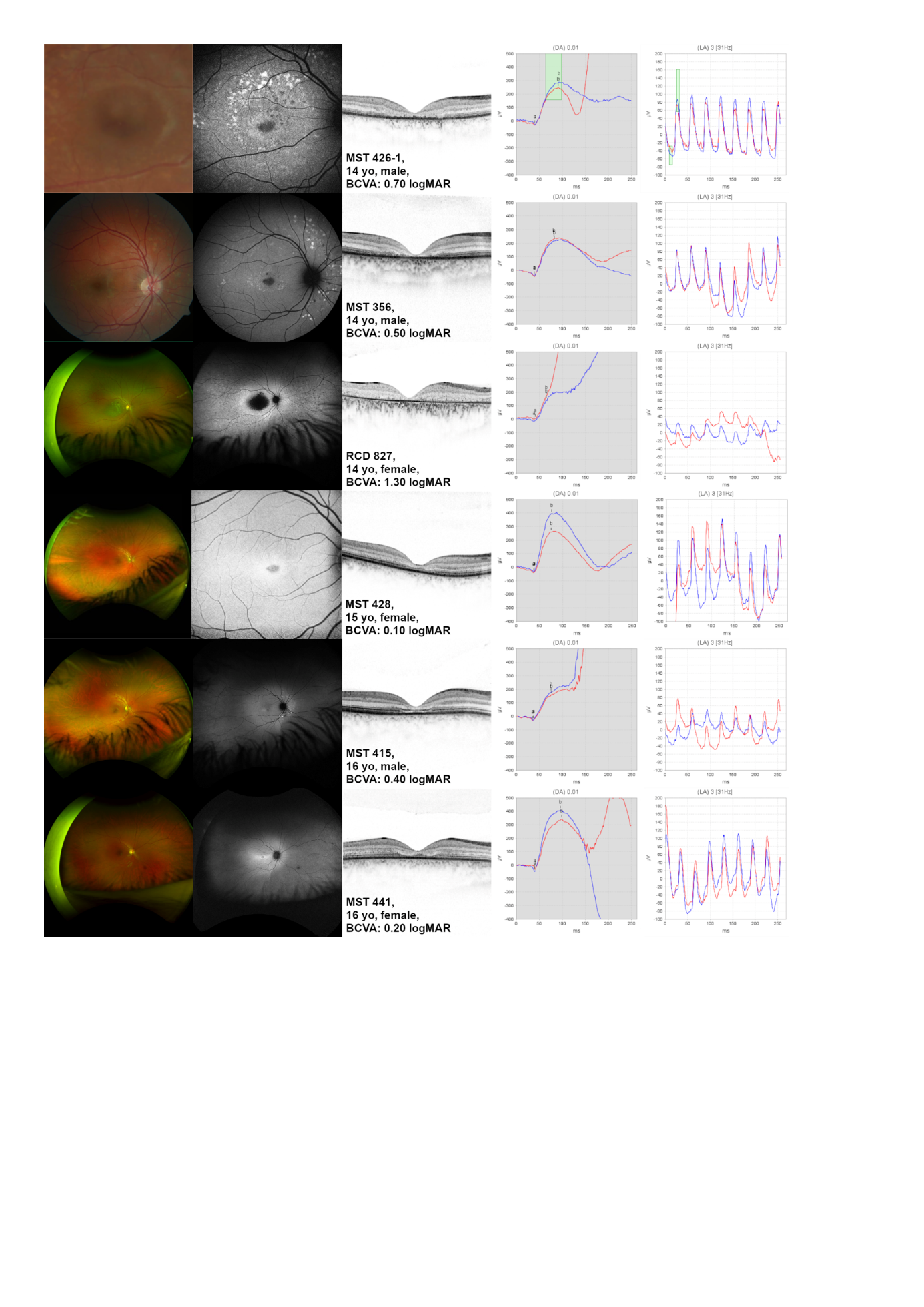


**Supplementary Table S1:** *ABCA4* variants and classification in this study.

| ***ABCA4* Variant** | **Number of alleles in this study** | **Literature (PMID)** | **MAF % gnomAD max/aggregated (cutoff 0.28% for PM2 and 0.42% for BS1)** | **Classification** | **Severity category according to Cornelis et al. 2023** | **dbSNP** |
| --- | --- | --- | --- | --- | --- | --- |
| **Missense** |  |  |  |  |  |  |
| c.52C>T;p.(Arg18Trp) | 1 | 9503029 | 0.0135%/0.0014% | Pathogenic | moderate | rs121909205 |
| c.214G>A;p.(Gly72Arg) | 5 | 10958763 | 0.0161%/0.0019% | Pathogenic | moderate | rs61751412 |
| c.634C>T;p.(Arg212Cys) | 2 | 9503029 | 0.0333%/0.0113% | Pathogenic | severe | rs61750200 |
| c.1253T>C;p.(Phe418Ser) | 1 | 21911583 | 0.007%/0.0051% | Likely pathogenic | severe | rs794726979 |
| c.1411G>A;p.(Glu471Lys) | 1 | 9295268 | 0.6248%/0.0725% | VUS | benign | rs1800548 |
| c.1622T>C;p.(Leu541Pro) | 5 | 9781034 | 0.0609%/0.013% | Pathogenic | severe | rs61751392 |
| c.1807T>C;p.(Tyr603His) | 2 | 29555955 | 0.0003%/0.0002% | Pathogenic | moderate | rs1064793006 |
| c.1957C>T;p.(Arg653Cys) | 1 | 10958763 | 0.0044%/0.0027% | Pathogenic | severe | rs61749420 |
| c.2894A>G;p.(Asn965Ser) | 2 | 9054934 | 0.0223%/0.0075% | Pathogenic | moderate | rs201471607 |
| c.2933G>A;p.(Gly978Asp) | 1 | 11702214 | n/a | Pathogenic | severe | rs61749453 |
| c.3113C>T;p.(Ala1038Val) | 5 | 9054934 | 0.5513%/0.1848% | Likely pathogenic | mild | rs61751374 |
| c.3259G>A;p.(Glu1087Lys) | 1 | 9054934 | 0.0066%/0.0024% | Pathogenic | severe | rs61751398 |
| c.3322C>T;p.(Arg1108Cys) | 2 | 9781034 | 0.0247%/0.0194% | Pathogenic | moderate | rs61750120 |
| c.3364G>A;p.(Glu1122Lys) | 2 | 9973280 | 0.0033%/0.0017% | Pathogenic | severe | rs61751399 |
| c.3701C>T;p.(Pro1234Leu) | 2 | 32531858 | 0.0029%/0.0003% | Likely pathogenic | moderate/severe | rs1383231039 |
| c.4462T>C;p.(Cys1488Arg) | 2 | 9973280 | 0.0056%/0.0009% | Pathogenic | severe | rs61750146 |
| c.4918C>T;p.(Arg1640Trp) | 1 | 9781034 | 0.0164%/0.0054% | Pathogenic | severe | rs61751404 |
| c.4919G>A;p.(Arg1640Gln) | 1 | 10711710 | 0.0067%/0.0018% | Pathogenic | severe | rs61751403 |
| c.5318C>T;p.(Ala1773Val) | 2 | 18652558 | 0.0067%/0.0025% | Pathogenic | severe | rs760549861 |
| c.5512C>G;p.(His1838Asp) | 2 | 23755871 | 0.0173%/0.0001% | Likely pathogenic | severe | rs62642562 |
| c.5603A>T;p.(Asn1868Ile) | 2 | 11328725 | 6.7328%/5.58% | VUS / known hypomorphic allele | mild | rs1801466 |
| c.5882G>A;p.(Gly1961Glu) | 4 | 23755871 | 2.2429%/0.3406% | Pathogenic | mild | rs1800553 |
| c.6077T>C;p.(Leu2026Pro) | 2 | 28118664 | 0.006%/0.0005% | Pathogenic | moderate | rs886044758 |
| c.6079C>T;p.(Leu2027Phe) | 1 | 9054934 | 0.0611%/0.0469% | Pathogenic | moderate | rs61751408 |
| c.6229C>T;p.(Arg2077Trp) | 1 | 9054934 | 0.0016%/0.0009% | Pathogenic | severe | rs61750645 |
| **Nonsense** |  |  |  |  |  |  |
| c.1903C>T;p.(Gln635Ter) | 1 | 10958763 | 0.001%/0.0005% | Pathogenic | severe | rs61749414 |
| c.1988G>A;p.(Trp663Ter) | 1 | 10958763 | 0.00025/0.0001% | Pathogenic | severe | rs865990202 |
| c.2041C>T;p.(Arg681Ter) | 1 | 10090887 | 0.0022%/0.0019% | Pathogenic | severe | rs61749423 |
| c.2626C>T/p.(Gln876Ter) | 1 | 34321860 | n/a | Pathogenic | severe | n/a |
| c.4234C>T;p.(Gln1412Ter) | 2 | 10090887 | 0.0032%/0.0019% | Pathogenic | severe | rs61750137 |
| **Splicing** |  |  |  |  |  |  |
| c.571-1G>T;p.(?) | 1 | 11702214 | 0.003%/0.0002% | Pathogenic | severe | rs61748533 |
| c.768G>T;p.(Val256=) | 1 | 20647261 | 0.0105%/0.0079% | Pathogenic | severe | rs62645944 |
| c.2588-7_2588-5delinsGG;p.(?) | 1 | 32531858 | n/a | VUS | uncertain | n/a |
| c.3329-1G>A;p.(?) | 2 | 31212395 | 0.0013%/0.0005% | Likely pathogenic | severe | rs544428779 |
| c.4254-1G>C;p.(?) | 1 | 31456290 | 0.0003%/0.0002% | Likely pathogenic | severe | rs886044740 |
| c.4849-2A>G;p.(?) | 2 | 37734845 | n/a | (Pathogenic) | n/a | n/a |
| c.5018+2T>C;p.(?) | 2 | 19074458 | 0.0011%/0.0008% | Pathogenic | severe | rs61750562 |
| c.5197-557G>T;p.(?) | 1 | 30670881 | n/a | Likely pathogenic | severe | n/a |
| c.5461-10T>C;p.(?) | 3 | 15614537 | 0.0707%/0.0535% | Pathogenic | severe | rs1800728 |
| c.5714+5G>A;p.(?) | 5 | 9466990 | 0.0516%/0.0416% | Pathogenic | moderate | rs61751407 |
| **Deletion/Duplication** |  |  |  |  |  |  |
| c.571_580dup;p.(Gly194ValfsTer89) | 2 | 32531858 | n/a | Pathogenic | severe | n/a |
| c.2829del;p.(Pro944GlnfsTer6) | 2 | 23882696 | n/a | Pathogenic | severe | n/a |
| c.3642_3644del;p.(His1215del) | 2 | 32531858 | n/a | Likely pathogenic | moderate | rs1570367398 |
| c.3664_3669del;p.(Val1222_Glu1223del) | 1 | 37734845 | n/a | VUS | n/a | rs1570367367 |
| c.5917del;p.(Val1973Ter) | 3 | 10958763 | 0.0165%/0.0014% | Pathogenic | severe | rs61751389 |

**Footnotes:** MAF, minor allele frequency; max - maximal MAF in gnomAD in a subpopulation; n/a, not applicable; VUS, variant of uncertain significance. ACMG classification and severity category as suggested by Cornelis and coworkers [23] according to ClinGen recommendations; If variant was not listed and assessed herein, ACMG classification was assessed using the web-based variant interpretation tool Franklin applying custom settings (Genoox Ltd, [https://franklin.genoox.com/Queries February 2024](https://franklin.genoox.com/Queries%20February%202024)), and classification is provided in brackets.
